# Human B Cells Mediate Innate Anti-Cancer Cytotoxicity Through Concurrent Engagement of Multiple TNF Superfamily Ligands

**DOI:** 10.3389/fimmu.2022.837842

**Published:** 2022-03-22

**Authors:** Bratislav M. Janjic, Aditi Kulkarni, Robert L. Ferris, Lazar Vujanovic, Nikola L. Vujanovic

**Affiliations:** ^1^ UPMC Hillman Cancer Center, University of Pittsburgh, Pittsburgh, PA, United States; ^2^ Department of Otolaryngology, University of Pittsburgh, Pittsburgh, PA, United States; ^3^ Department of Immunology, University of Pittsburgh, Pittsburgh, PA, United States; ^4^ Department of Pathology, University of Pittsburgh, Pittsburgh, PA, United States

**Keywords:** B cells, cytotoxic, innate immunity, TNF superfamily ligands, cancer, head and neck squamous cell carcinoma, single-cell RNA sequencing (scRNAseq)

## Abstract

The essential innate immunity effector cells, natural killer and dendritic cells, express multiple plasma membrane-associated tumor necrosis factor (TNF) superfamily (TNFSF) ligands that, through simultaneous and synergistic engagement, mediate anti-cancer cytotoxicity. Here, we report that circulating B cells, mediators of adaptive humoral immunity, also mediate this innate anti-cancer immune mechanism. We show that resting human B cells isolated from peripheral blood induce apoptosis of, and efficiently kill a large variety of leukemia and solid tumor cell types. Single-cell RNA sequencing analyses indicate, and flow cytometry data confirm that B cells from circulation express transmembrane TNF, Fas ligand (FasL), lymphotoxin (LT) α1β2 and TNF-related apoptosis-inducing ligand (TRAIL). The cytotoxic activity can be inhibited by individual and, especially, combined blockade of the four transmembrane TNFSF ligands. B cells from tumor-bearing head and neck squamous cell carcinoma patients express lower levels of TNFSF ligands and are less cytotoxic than those isolated from healthy individuals. In conclusion, we demonstrate that B cells have the innate capacity to mediate anti-cancer cytotoxicity through concurrent activity of multiple plasma membrane-associated TNFSF ligands, that this mechanism is deficient in cancer patients and that it may be part of a general cancer immunosurveillance mechanism.

## Introduction

Previous studies have determined that human and mouse natural killer (NK) cells and dendritic cells (DCs) constitutively express multiple transmembrane cytotoxic tumor necrosis factor (TNF) super family (TNFSF) ligands, including TNF, FasL, LT-α1β2 and/or TRAIL. By simultaneous engagement of these ligands, NK cells and DCs mediate a potent cytotoxic mechanism that efficiently induces apoptosis in virtually all types of hematologic and solid tissue cancer cells without affecting non-cancerous cells (1–5). This innate cytotoxic activity might be an important immunosurveillance mechanism and a potential target for cancer prevention and therapy.

The adaptive immune system is constituted of highly heterogeneous populations of B and T cells that mediate the well-known antigen specific immune functions, but also the less appreciated innate immune functions (6–12). B cells, in particular, can respond to and eliminate foreign antigens by producing specific antibodies, but also can produce immunostimulatory and immunosuppressive cytokines and regulate immune responses (6–8, 10). Additionally, B cells express MHC class II molecules and are potent antigen presenting cells that process and present antigens to antigen-specific B and T cells, and constitutively express on their plasma membrane LT-α1β2, the TNFSF ligand complex made up of one molecule of LT-α and two transmembrane molecules of LT-β, that mediates development of the secondary lymphoid tissue architecture by interacting with the stromal cell LT-β receptor (LTβR) (13). Furthermore, it has been reported that activated B cells express several other TNFSF ligands, including TNF, Fas ligand (FasL), TRAIL, OX40L, CD30L, CD40L CD70, LIGHT, GITRL, BAFF and APRIL (14–17) and, therefore, they might be capable of mediating various innate immune functions, including killing of cancer cells. This notion is supported by previous studies that have suggested that activated B cells possess tumoricidal potential that might be mediated by a number of mechanisms, including TNFSF ligands and granzyme B (7, 14, 15, 18).

In the present study, we newly determined that human resting B cells express on their plasma membrane TNF, FasL, LT-α1β2 and TRAIL, and mediate apoptosis of a broad range of leukemia and solid tissue cancer cells *via* simultaneous activity of these transmembrane TNFSF ligands, while mostly sparing normal cells. We also found that circulating B cells isolated from head and neck squamous cell carcinoma (HNSCC) patients express lower levels of TNFSF ligands and are less cytotoxic than those from healthy individuals. These findings indicate that B cells may have a significant role in innate cancer immunosurveillance.

## Materials and Methods

### Blood Donors and Peripheral Blood Leukocyte (PBL) Isolation

Blood of treatment-naive HNSCC patients was obtained with an IRB (HCC #99-069)-based informed consent. Healthy donor buffy coats were acquired from the Central Blood Bank. PBL were separated from blood or buffy coats using Ficoll-Paque PLUS gradient centrifugation (Cytiva; Marlborough, MA) as previously described (19).

### Reagents

The following fluorescence-conjugated monoclonal antibodies (mAbs) were applied in this study: anti-CD19 (FITC, clone 4G7, IgG1; BioLegend; San Diego, CA), anti-CD3 (Alexa Fluor 700 or PE-Cy7, clone HIT3a, IgG2b; BioLegend), anti-CD4 (PerCP-Cy5.5, clone OKT4, IgG2b; BioLegend), anti-CD14 (PE, clone M5E2, IgG2a; BD Biosciences; Franklin Lakes, NJ), anti-CD56 (APC, clone HCD56, IgG1; BioLegend), anti-TNF (Alexa Fluor 647, clone 4G7, IgG1; BioLegend), anti-CD178/FasL (BUV395, clone NOK-1, IgG1; BD Biosciences), anti-CD253/TRAIL (BV711, clone RIK-2, IgG1; BD Biosciences), anti-TNF-β/LT-α (Alexa Fluor 405, clone 15A3, IgG2b; Novus Biologicals; Littleton, CO) and anti-LT-β (PE, polyclonal, IgG; Biorbyt; St. Louis, MO). Appropriate isotype controls were acquired from the corresponding vendors. We also used human recombinant IL-2 (Chiron, Emeryville, CA), and the following human dimeric fusion proteins of TNFSF receptors and Fc IgG1 fragment: TNFRp60:Fc, LTβR:Fc (Biogen, Cambridge, MA), TNFRp80:Fc (Immunex, Seattle, WA), Fas : Fc (R&D Systems, Minneapolis, MN), and TRAILR2:Fc (Alexis Biochemicals, San Diego, CA); as well as human IgG1 kappa (SIGMA-ALDRICH, St. Louis, MO) and the human dimeric fusion protein IL-4 receptor and Fc IgG1 fragment (IL-4R:Fc) (Immunex).The reagents from Immunex and Biogen were generous gifts. The T cell mitogen Concavalin-A, Etoposide (VP-16) and the pan-caspase inhibitor Z-VAD-FMK were obtained from Sigma-Aldrich. Caspase-8 inhibitor Z-IETD-FMK was obtained from R&D Systems.

### Generation of Immature DCs in Culture

Monocyte-derived immature DCs (iDCs) were generated as previously described (3, 19). Briefly, peripheral blood monocytes were purified by CD14 isolation (Miltenyi Biotec; Auburn, CA) and cultured for 7-day in the presence of recombinant human 800 IU/mL GM-CSF and 500 IU/mL IL-4 (both kindly provided by Schering-Plough Research Institute, Kenilworth, NJ).

### Purification of B cells and T cells

B and T cells were purified from healthy human donor or HNSCC patient PBL using the Miltenyi human Pan B cell and Pan T cell isolation kits, respectively (Miltenyi Biotec), according to the company protocols. Purified B and T cells were ≥90% CD19^+^CD3^-^CD56^-^CD14^-^ (**Supplementary Figure 1**) and more than 90% CD3^+^CD56^-^CD14^-^CD19^-^, respectively (data not shown). Among the purified CD19^+^ B cells, 38% expressed CD27 (data not shown).

### T Cell Activation and Culture

Purified CD3^+^ T cells (0.5 x 10^6^/mL) were suspended in RPMI-1640 medium, supplemented with 10% FCS (ThermoFisher Scientific; Waltham, MA), 10 μg/mL Concavalin-A and 0.22 nM (60 IU/mL) IL-2, seeded in T75 tissue culture flasks (BD Biosciences), and cultured for 4 days. At the end of this culture, activated T cells were 98% CD3^+^CD56^-^CD14^-^CD19^-^ (data not shown).

### Cell Lines

All cell lines were of human origin. They were cultured at 37°C in 5% CO_2_, in air, using RPMI-1640 or DMEM media supplemented with 10% FBS, 2 mM L-glutamine, 100 IU/ml penicillin, and 100 pg/ml streptomycin (ThermoFisher Scientific). Normal cell targets included T cell blasts (purified T cells stimulated for 4 days with 10 μg/ml Concavalin-A and 0.22 nM IL-2), dermal fibroblasts (NHDF-Ad), epidermal keratinocytes (NHEK-Ad) (Clonetics, BioWhittaker, Walkersville, MD), and HUVEC (Cell Systems, Kirkland, CA). Leukemia cell lines used in this study were Molt-4 acute lymphoblastic T cell leukemia, K562 myeloid leukemia, Jurkat T cell leukemia and Daudi Burkitt’s B cell lymphoma (American Type Culture Collection [ATCC], Manassas, VA). Solid tumor-derived cell lines included: HNSCC PCI-13, PCI-4A, PCI-4B, PCI-15A, PCI-15B, PCI-6A, PCI-6B, PCI-22A, PCI-22B, PCI-30 and PCI-37A (UPMC Hillman Cancer Center [HCC], Pittsburgh, PA); breast carcinomas BT20, MCF7, and SKBR3 (ATCC); melanomas FemX (ATCC), PMel136.34 and PMel255.1 (HCC); colon carcinomas LS174 and LS180 (NeoRx, Seattle, WA); lung squamous cell carcinomas LC226, LC358 and LC596 (HCC); lung small cell carcinomas LCH69 and LCH345 (HCC); gastric carcinoma HR (HCC); renal cell carcinoma RCC (HCC); ovarian carcinomas SKOV3, CAOV3, and OVCAR3 (ATCC); and gliomas P303, P319 (HCC) and SNB19 (National Institutes of Health, Bethesda, MD). The cell lines were negative for mycoplasma contamination as shown by GEN-PROBE Mycoplasma Tissue Culture Non-Isotopic Rapid Detection System (Gen-Probe, Inc.; San Diego, CA).

### ^51^Cr Release Assay

Target cells were plated in complete medium (5,000 cells per well) in a 96-well flat-bottom plate (Corning Inc., Corning, NY) 24-h prior to the assay. The following day, target cells were labeled with 100 µM chromium-51 radionuclide (New England Nuclear, Boston, MA). Effector cells were added to target cells at 3:1, 1:1, 0.3:1 and 0.1:1 effector to target (E:T) ratios and co-incubated at 37°C for 1, 4 or 24 h. Controls used for this assay were target cells alone, either in cell culture medium (spontaneous release) or in 5% Triton X-100 (Sigma-Aldrich; maximal release). After incubation, ^51^Cr was measured in the cell-free supernatants using a gamma counter. The percentage of cytotoxicity was determined using the formula 100 X (E-S)/(M-S), where E = experimental (CPM after co-culture of effectors and targets), S = spontaneous and M = maximal release.

### ^3^H-Thymidine Release Assay

Target cells were seeded as described for the ^51^Cr release assay 24-h prior to the assay. The next day, target cells were labeled with 5 µCi/mL of methyl ^3^H-thymidine (New England Nuclear; 147.9 GBq/mmol). Effector cells were added to target cells at 3:1, 1:1, 0.3:1 and 0.1:1 E:T ratios and co-incubated for 1-h at 37°C. Afterwards, cells were lysed by three rounds of freeze-thawing. The disrupted cells were harvested onto fiberglass filters, which were subsequently dried, immersed in liquid scintillation fluid, and their radioactivity was measured in an LKB Betaplate counter (Pharmacia, Gaithersburg, MD). The percentage of cytotoxicity was determined using the following formula: 100 X (C - E)/C, where E = experimental (cpm of target cells in the presence of effector cells) and C = control (cpm of target cells alone). Both C and E were cpm of the retained, insoluble (i.e., undamaged), ^3^H-thymidine-labeled DNA. Spontaneously developed and B cell-induced soluble fragments of ^3^H-thymidine labeled DNA were washed out during cell harvesting.

### MTT Cytotoxicity Assay

Target cells were plated as described for the ^51^Cr release assay 24-h prior to the assay. The following day, media was carefully removed from the wells and B cells resuspended in the same cell culture medium were plated on top of tumor cells at different E:T ratios. Control wells contained the medium (background), tumor (spontaneous death) or B cells (effector cell background) alone. Cells were co-incubated at 37°C for 3-24 h. Afterwards, the media was carefully pipetted out. 100 μL of 0.5 mg/mL MTT (3-[4,5-dimethylthiazole-2-yl]-2,5-diphenyltetrazolium bromide) solution in cell culture medium was added to the wells. After a 3-h incubation at 37°C, MTT solution was removed and 150 μL of 99.9% isopropanol (Sigma-Aldrich) was added to the wells. The plate was gently shaken for 20-30 min at room temperature and examined on the Epoch microplate spectrophotometer (BioTek; Winooski, VT) using excitation and emission wavelengths of 570 nm and 490 nm, respectively. The percentage of cytotoxicity was calculated using the following formula: % cytotoxicity = 100 − (E − BGEC)/(TS − BG0) × 100, where BG0 is background of medium alone, TS is total viability/spontaneous death of untreated target cells, BGEC is background of effector cells, and E is experimental well. For caspase inhibition experiments, pan caspase (Z-VAD-FMK) and caspase-8 (Z-IETD-FMK) inhibitors were used at 50 µM and 100 µM working concentrations, respectively. The inhibitors were added to plated PCI-13 targets 1-h prior to the addition of healthy donor B cells at 1:1 E:T ratio. For TNFSF ligand blocking experiments, 10 µg/mL of TNFSF receptor-Fc fusion proteins specific for TNF (TNFR1:Fc), FasL (Fas : Fc), TRAIL (TRAILR : Fc) and LT-α1β2 (LTβR:Fc), as well as IgG isotype and IL-4R:Fc fusion protein controls were added to appropriate wells 1-h prior to adding B cells. For these experiments, B cells were added at 1:1 E:T ratio. For the assessment of the role of cell-to-cell contact or of the need for cell secretion in B cell-mediated tumor cell killing, healthy donor B cells were fixed with 1% w/v paraformaldehyde in 1X PBS for 15 min at room temperature. Subsequently, fixed cells were washed three times, resuspended in the complete medium and added at 3:1 E:T ratio. Experiments with cytotoxicity assays were performed in 4 to 6 replicates per each E:T ratio.

### Flow Cytometry

Cells were washed and resuspended in FACS buffer (PBS + 0.2% w/v BSA + 0.02% w/v NaN3). For TNFSF ligands, cells were labeled with 20 µg/ml of each antibody or matching isotype control. Staining was performed at room temperature for 30 min in the dark, after which cells were washed twice in FACS buffer and resuspended in BD Cytofix (BD Biosciences). Antibodies against lineage markers were used per manufacturers’ instructions. Flow cytometry data was acquired using the BD LSRFortessa II analyzer, BD Accuri™ C6 (BD Biosciences) and CytoFlex LX (Beckman Coulter; Brea, CA). For TNFSF ligand evaluation, 10,000 CD19^+^CD3^-^ B cells were acquired. Fluorescence minus five (FM5) controls labeled with CD3 AF700 and CD19 FITC, as well as control IgG antibodies for AF647, AF405, PE, BUV395 and BV711 antibodies, were used to set upper limits for background signals on the missing labels. Data were analyzed using FlowJo v10 (FlowJo, LLC; Ashland, OR).

### Statistical Analysis

The cytotoxicity was presented as the means of cytotoxicity percentages for each E:T ratio and LU_20_/10^7^ effector cells. LU_20_/10^7^ effector cells were determined using the formula 10^7^/(T x X_20_), where T is the number of target cells and X_20_ is the estimated E:T ratio at which 20% of the target cells were killed. Data are presented as mean values ± standard error of means (SEM), and as box plots with medians, quartiles, and ranges. Statistical significance of differences of results were assessed using the Student’s *t*-test, and one- and two-way ANOVA test. Differences were considered significant when the value of *P* was ≤ 0.05.

### Single-Cell RNA Sequencing (scRNAseq) Data Availability

Unprocessed FASTQ files for our published scRNAseq datasets (20, 21) are available on NCBI Sequence Read Archive (BioProject ID PRJNA579178 and PRJNA691564). Processed gene barcode matrices are available on the Gene Expression Omnibus database (Accession ID GSE139324 and GSE164690). Demographics of 29 HNSCC patients evaluated in these studies is summarized in **Supplementary Table 1**.

### Processing, Clustering, Cell Type Identification and Quantification of Differences in scRNAseq

Normalization, dimensionality reduction and data visualization of scRNAseq data was performed using the methodology previously described (20–22). Subsequently, a neighborhood graph was constructed to identify related groups of cells, after which Leiden clustering was performed on the neighborhood graph. The cluster assignments were then visualized on Uniform Manifold Approximation and Projection (UMAP) plots (23–26). Using a combination of top expressed genes in each cluster and a list of known marker genes, cell types were assigned to each cluster. Analysis of variance was used to determine if the gene expression across B cell groups was statistically significant, and Wilcoxon rank sum tests were used to determine if there were statistically significant differences in cell frequencies and gene expression levels between healthy donor (HD) and HNSCC patient samples.

## Results

### Resting B Cells Induce Apoptosis in Cancer Cells

**Figure 1:**
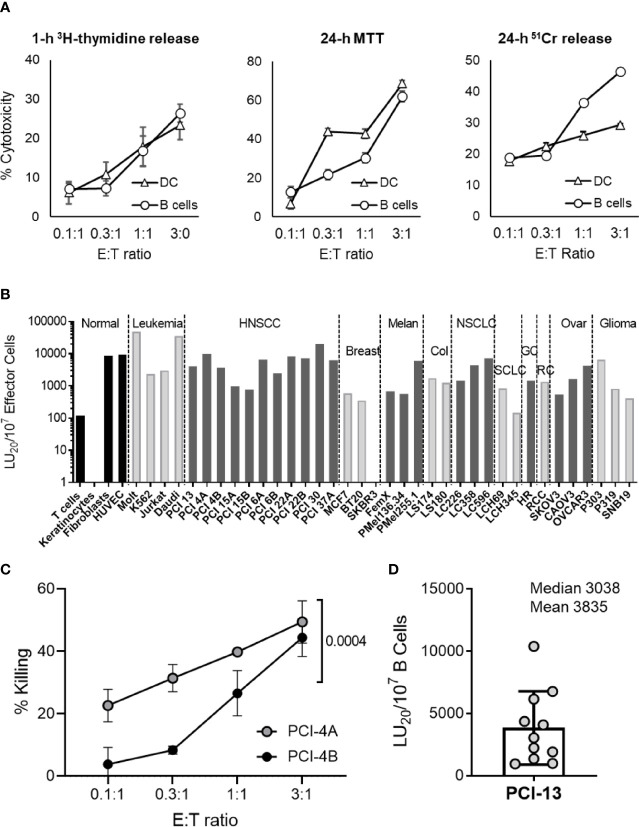
Resting B cells isolated from peripheral blood induce apoptosis in a broad panel of different types of cancer cells. **(A)** Resting healthy donor B cells were compared to iDCs for their ability to induce apoptosis in cancer cells. MACS-purified human peripheral blood resting B cells (B cells) and *in vitro*-generated iDCs were co-incubated with PCI-13 target cells at the indicated E:T ratios and tested for their cytotoxic activity using 1-h ^3^H-thymidine release, 24-h MTT and 24-h ^51^Cr release assays. Each test was performed in quadruplicates and mean percent cytotoxicity values ± standard deviations (STDEV) are shown. Data are representative of three independent experiments. **(B)** The susceptibility of normal and cancer cell lines to cytotoxic activity of resting B cells was tested using 1-h ^3^H-thymidine release assay (non-adherent leukemia and healthy donor T cell blast cells) and 24-h MTT assays (adherent cell monolayers). Data are presented as LU_20_/10^7^ effector cells based on the mean % cytotoxicity values of quadruplicate tests obtained at four different E:T ratios. HNSCC cell lines labeled with “A” or “B” originated from primary and metastatic lesions, respectively. The cytotoxicity against various targets was tested 1, 2 or, in the case of PCI-13, 11 times. The presented data are from representative experiments. NORMAL, normal cell lines; LEUKEMIA, leukemia cell lines; HNSCC, squamous cell carcinoma of head and neck cell lines; BREAST, breast carcinoma cell lines; MELAN, melanoma cell lines; COL, colorectal carcinoma cell lines; NSCLC, non-small cell lung carcinoma cell lines; SCLC, small cell lung carcinoma cell lines; GC, gastric carcinoma cell line, RC, renal carcinoma cell line; OVAR, ovarian carcinoma cell lines; GLIOMA, glioma cell lines. **(C)** Paired primary tumor (PCI-4A) and lymph node metastasis (PCI-4B) cancer cells obtained from an HNSCC patient were tested in parallel for their susceptibility to killing by resting B cells using 24-h MTT assay. Mean % cytotoxicity values and STDEV obtained from quadruplicate tests and at four E:T ratios (0.1:1, 0.3:1, 1:1 and 3:1) are plotted. Two-way ANOVA was used to calculate the differences between groups. Data are representative of two independent experiments. **(D)** Resting B cells isolated from 11 healthy blood donors were tested for their cytotoxic activity against PCI-13 target cells using the 24-h MTT assay. Data are presented as LU_20_/10^7^ B cells based on the mean % cytotoxicity values of quadruplicate tests obtained at four E:T ratios.

Previously, we have determined that the major innate immunity effector cells, NK cells and DCs, potently kill cancer cells by inducing apoptosis without MHC restriction (1, 3). To expand on these observations, we tested whether the circulating B cells were also capable of killing cancer cells and whether they did so by inducing apoptosis. This was examined by comparatively testing the ability of healthy donor peripheral blood resting B cells and monocyte-derived iDCs to kill PCI-13 HNSCC target cells using the apoptosis specific assays 1-h ^3^H-thymidine release, 24-h MTT (**Figure 1A**) and 3-h MTT (**Supplementary Figure 2A**). B cell cytotoxicity was confirmed using the 24-h ^51^Cr release assay (**Figure 1A**). In all three assays, DCs and resting B cells displayed efficient and dose-dependent killing of cancer cells at low effector-to-target (E:T) ratios (0.3:1, 1:1 and 3:1). In addition, B cell-mediated cytotoxic activity was significantly inhibited with the pan-caspase inhibitor Z-VAD-FMK (**Supplementary Figure 2B**) and caspase 8 inhibitor Z-IETD-FMK (**Supplementary Figure 2C**). In sharp contrast, necrosis of tumor cells was not observed in 1-h and 4-h ^51^Cr release assays (**Supplementary Figure 2D**). Therefore, similar to DCs, resting B cells are capable of inducing apoptosis in cancer cells.

### B Cells Kill a Broad Range of Hematologic and Solid Tissue Cancer Cells

Resting circulating B cells were assessed for their ability to kill four different non-malignant cell types, 4 different leukemia cell lines and 32 different carcinoma cell lines using 24-h MTT and/or 1-h ^3^H-tymidine release assays (**Figure 1B**). With the exception of SKBR3 breast carcinoma and LCH345 small cell lung carcinoma, resting B cells efficiently killed 34 of 36 tested cancer cell lines, with Molt-4 (T-cell leukemia) and Daudi (Burkitt B-cell lymphoma) cell lines being the most efficiently killed. Similarly to DCs (3), B cells killed primary HNSCC tumor cell lines more efficiently than patient-matched lymph node metastasis cell lines (**Figure 1C**, **Supplementary Figure 3** and **Supplementary Table 2**). In contrast to cancer cell targets, resting B cells did not readily kill T cell blasts and keratinocytes, but did efficiently kill proliferating fibroblasts and endothelial cells. These data demonstrate that resting B cells, similar to NK cells and DCs (1–5), possess the innate anticancer cytotoxic mechanism that is mediated against many types of cancer cells and some types of non-malignant cells.

### B Cell Tumoricidal Activity Is Omnipresent in Healthy Individuals

To determine whether the observed apoptosis-inducing anticancer activity is a common physiological mechanism of resting circulating B cells, we analyzed the killing ability of these cells obtained from the peripheral blood of 11 healthy donors (**Figure 1D**). We found that resting B cells from all evaluated individuals were cytotoxic against the prototypical PCI-13 HNSCC target cells. The efficiency of this cell-mediated cytotoxic activity was donor-dependent. These data show that the tumoricidal activity is an essential function of healthy human resting B cells and indicate that it may be driven by genetic and/or environmental factors.

### Resting B Cells Express Multiple Cytotoxic TNFSF Ligands

**Figure 2:**
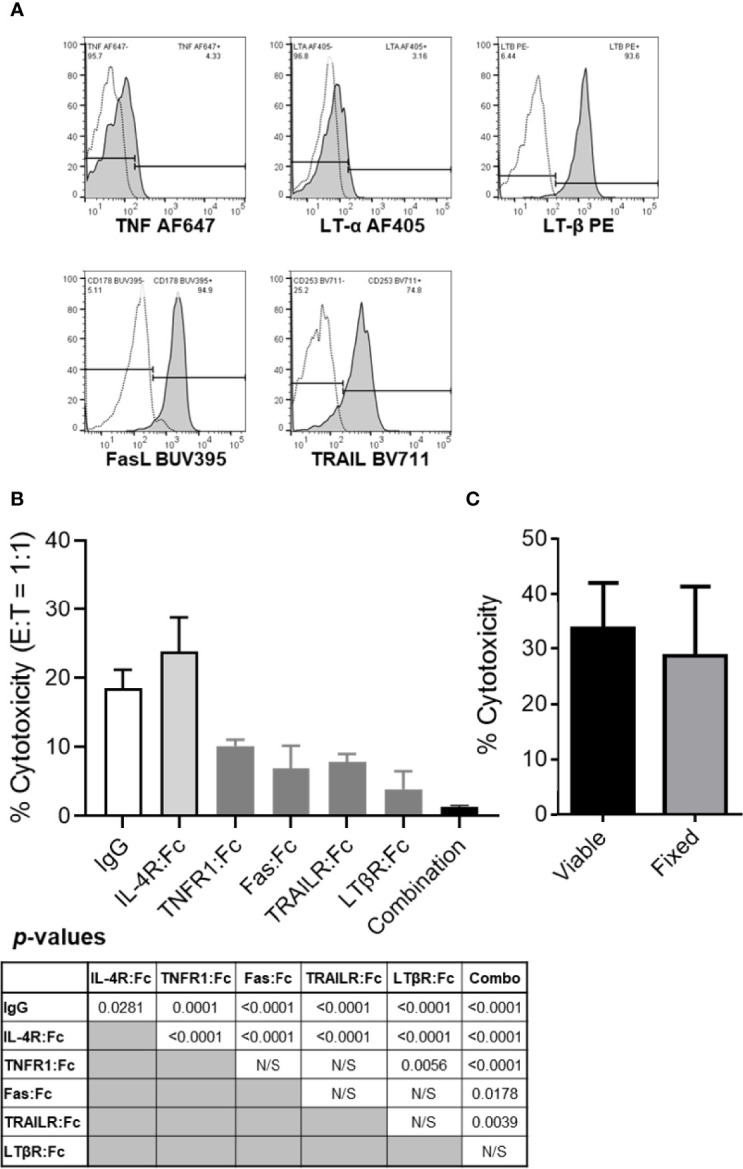
The tumor killing ability of resting B cells is inhibited by TNF, FasL, TRAIL and/or LT-α1β2 blockades but not by cellular fixation with paraformaldehyde. **(A)** Healthy donor PBL were stained for flow cytometry per *Materials and Methods*. Lymphocytes were gated based on their FSC vs SSC profile and single-cell status and, subsequently, evaluated for CD3 and CD19 expression. CD19^+^CD3^-^ B cells were analyzed for surface TNF, LT-α, LT-β, FasL and TRAIL expression levels. Representative data from one of the four healthy donors evaluated are shown. Empty histograms represent IgG controls, and filled histograms represent the TNFSF ligand staining. **(B)** TNFSF receptor-Fc fragment fusion proteins were used to block healthy donor B cell cytotoxicity of PCI-13 cells at 1:1 E:T ratio. Where indicated, effector cells were preincubated with 10 μg/mL of the individual or combined (combination) TNFSF receptor-Fc fusion proteins specific for TNF (TNFR1:Fc), FasL (Fas : Fc), TRAIL (TRAILR : Fc) and LT-α1β2 (LTβR:Fc) for 1h at 37°C. Controls included IgG1 isotype and IL-4R:Fc fusion protein controls. Following preincubation with antagonistic agents, the cytotoxic activity of B cells was assessed using the 3-h MTT assay. Data represent the mean % cytotoxicity of 6 replicates ± STDEV, and statistical significance was calculated using the one-way ANOVA. Calculated *p*-values are listed in the summary table below the figure. A representative of 3 experiments is shown. **(C)** Resting healthy donor B cells fixed with 1% paraformaldehyde were compared to their viable counterparts for their ability to kill PCI-13 cells. The cytotoxic activity of B cells was assessed using the 24-h MTT assay at 3:1 E:T ratio. Data represent the mean % cytotoxicity of 4 replicates ± STDEV, and statistical significance was calculated using the two-tailed Student’s *t*-test. A representative of 2 experiments is shown.

It has been shown that resting B cells express LT-α1β2 and that activated B cells can upregulate TNF, FasL and TRAIL and several other TNFSF ligands (13–17). It is unclear whether resting human B cells can also express plasma membrane-associated cytotoxic TNFSF ligands TNF, FasL and TRAIL. We evaluated whether resting healthy donor peripheral blood B cells express the five plasma membrane-associated proteins that constitute the four TNFSF ligand complexes previously associated with NK cell- and DC-mediated cancer cell killing (2, 4). PBL were simultaneously stained for surface expression of CD3 (pan-T cell marker), CD19 (pan-B cell marker) and TNFSF ligands. Flow cytometric analysis of stained healthy donor PBL showed that CD19^+^CD3^-^ B cells expressed relatively low levels of plasma membrane-associated TNF and LT-α, and high levels of LT-β, FasL and TRAIL (**Figure 2A**).

### Antagonists of TNFSF Ligands Inhibit Cell Contact-Mediated B Cell Killing of Cancer Cells

Next, we tested whether the expressed TNFSF ligands mediate the apoptotic killing of cancer cells by resting healthy donor B cells (**Figure 2B**). These experiments were performed using the 3-h MTT assay with PCI-13 HNSCC target cells that express all the TNFSF receptors that can be triggered by the four TNFSF ligand complexes expressed on resting B cells (5). The assessment of TNFSF ligand blockade on resting B cell-mediated cytotoxicity was effectuated by preincubation of the effector cells with individual or combined TNFR1:Fc, Fas : Fc, TRAILR2:Fc and LTβR:Fc fusion proteins, or control reagents, human IgG1 and IL-4:Fc fusion protein, and by measuring the effector cell cytotoxic activity at 1:1 E:T ratio. We found that the individual antagonists inhibited 57% to 84%, while the four combined chimeric TNFSF receptors inhibited 95% of cytotoxic activity of resting B cells (**Figure 2B**). To assess whether B cells induce cancer cell killing *via* secreted or transmembrane molecules we examined the cytotoxic activity of paraformaldehyde-fixed B cells and found that they can kill cancer cells as effectively as their viable counterparts at 3:1 E:T ratio in the 24-h MTT assay (**Figure 2C**). As fixed cells are unable to actively secrete soluble factors, these data indicate that B cells utilize transmembrane molecules to kill tumor cells. Cumulatively, these data show that most, if not all of resting B cell tumoricidal activity is mediated by a simultaneous engagement of transmembrane TNF, FasL, TRAIL and LT-α1β2, that each of the four cytotoxic TNF family ligand complexes is an essential contributor to this activity and that cell-to-cell contact and transmembrane molecules, but not secreted soluble molecules, are required for this killing mechanism.

### High-Dimensional scRNAseq Data Analysis of TNFSF Ligand Expression Patterns in Healthy Donor and HNSCC Patient Peripheral Blood B cells

**Figure 3:**
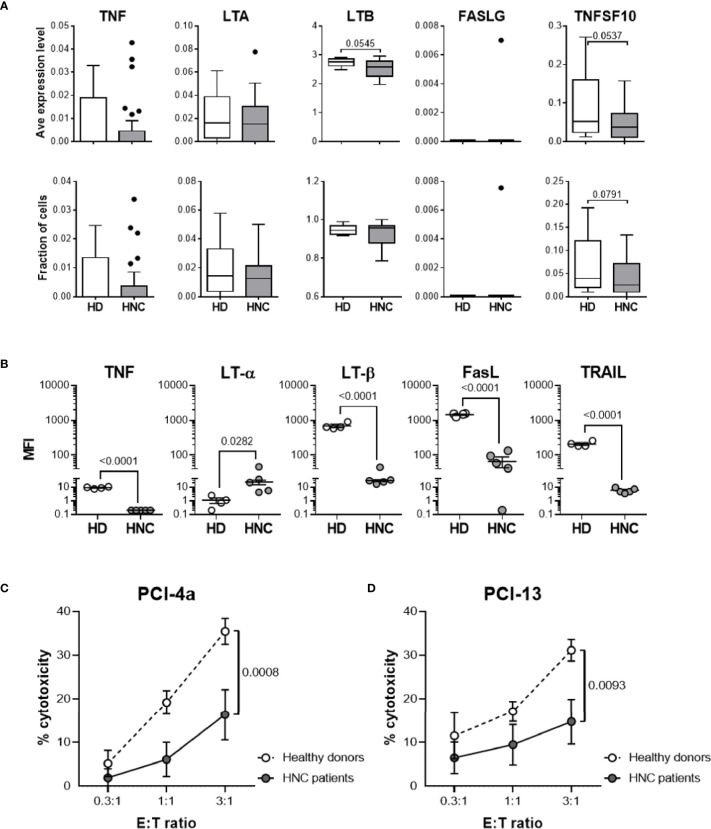
HNSCC patient circulating B cells display decreased levels of TNFSF ligand expression and tumoricidal activity. **(A)** Using our published scRNAseq datasets (20), we performed an analysis of healthy donor (HD; n = 6) and HNSCC patient (HNC; n = 29) PBL using the clustering strategy depicted in **Supplementary Figure 4**. Donor-specific relative expression levels of and fraction of B cells expressing TNFSF ligand mRNA are presented using box plots with medians, quartiles, and ranges (healthy donor PBL, HD - PBL; HNC patient PBL, HNC – PBL). **(B)** Resting B cells obtained from peripheral blood of 4 healthy donors (HD-PBL) and 5 HNSCC patients (HNC-PBL) were examined for the expression of cell surface TNFSF ligand proteins using multi-color flow cytometry with the specific antibodies for cell markers (B cell CD19; T cell CD3) and for TNFSF ligands (TNF, LT-α, LT-β, FasL, and TRAIL) as described in **Supplementary Figure 5**. To avoid possible batch effects, all donor samples were simultaneously stained and analyzed using the same procedure. Data represent donor-specific MFI values for each TNFSF ligand adjusted for respective IgG controls. Intersecting lines represent median MFI values of each donor cohort. *p*-values calculated using the one-tailed Student’s *t*- test are indicated in **(A, B)**. **(C, D)** MACS-purified circulating resting B cells were obtained from 4 healthy donors (HD) and 4 tumor-bearing untreated HNSCC patients (HNC), added to the monolayers of **(C)** PCI-4a and **(D)** PCI-13 HNSCC target cells at indicated E:T ratios and in quadruplicates, and comparatively tested for cytotoxic activity using the 24-h MTT assay. Data represent mean % cytotoxicity ± SEM of 4 healthy donors and 4 HNSCC patients. Individual donor-specific B cell cytotoxicity is shown in **Supplementary Figure 6**. Statistical significance was determined using the 2-way ANOVA test.

Using our published scRNAseq datasets (20, 21), we set out to investigate and compare the TNFSF ligand profiles of peripheral blood B cells isolated from healthy donors and HNSCC patients. The dataset included 147,562 immune (PBL and tumor-infiltrating leukocytes) and 37,909 non-immune cells isolated from 29 treatment-naïve patients and 6 healthy donors. These data are visualized as a comprehensive UMAP plot (**Supplementary Figure 4A**) (23–26). Subsequently, we extracted, subclustered, and visualized the transcriptomic profiles of 16,126 cells identified as B and plasma cells using canonical B cell lineage markers *CD19* and *MS4A1* (CD20) and plasma cell markers *SDC1* (CD138) and *TNFRSF17* (B cell maturation protein; **Supplementary Figure 4A**) (22). To specifically analyze TNFSF ligand expression by circulating B cells, we excluded clusters 5, 6, 8 and 11 that contain plasma cells based on low expression levels of CD19 and MS4A1 and high expression levels of SDC1 and TNFRSF17 (**Supplementary Figure 4B**). After removing tumor-infiltrating B cells from the analysis, 3,426 B cells from circulation with 941 median genes per cell were retained, with healthy donors contributing 678 and HNSCC patients contributing 2,748 B cells to the dataset (**Supplementary Figure 4A**). Subsequently, we focused on characterizing *TNF*, *LTA* (LT-α), *LTB* (LT-β), *FASLG* (FasL) and *TNFSF10* (TRAIL) expression by healthy donor- and HNSCC patient-associated circulating B cells (**Figure 3A**). We found that, with the exception of *FASLG*, circulating B cells expressed all the TNFSF ligand transcripts. Furthermore, we observed that relative expression levels of *TNF*, *LTB* (P = 0.0545) and *TNFSF10* (*P* = 0.0537) in HNSCC patient B cells were lower than those in healthy donors.

### TNFSF Ligand Expression on Circulating B Cells Is Decreased in HNSCC Patients

Our scRNAseq data suggested that HNSCC patient B cells express decreased levels of TNFSF ligands when compared to those from healthy donors. To validate these observations, we tested whether peripheral blood resting B cells isolated from healthy donors and treatment-naïve, tumor-bearing HNSCC patients differentially expressed the five plasma membrane proteins that constitute the four cytotoxic TNFSF ligand complexes (**Figure 3B**). Multicolor flow cytometric analysis was performed as described in **Figure 2** and **Supplementary Figure 5**. CD19^+^CD3^-^ healthy donor B cells expressed low levels of membrane-associated TNF and LT-α and relatively high levels of LT-β, FasL and TRAIL. In comparison, peripheral blood B cells from HNSCC patients displayed significantly decreased levels of TNF, LT-β, Fas-L and TRAIL, and significantly increased levels of LT-α on their surface (**Figure 3B** and **Supplementary Figure 5**). Therefore, resting healthy donor B cells express the four cytotoxic TNFSF ligand complexes previously shown to be presented and functional on NK cells and DCs (3, 4). Furthermore, expression of all these ligands, with the exception of LT-α is significantly diminished on HNSCC patient B cells, similar to what was previously reported for NK cells and DCs (27). Decreased LT-β expression and concurrent increased LT-α expression on B cells may indicate that in cancer patients the canonic LT-α1β2 ligand complex that reacts with LT-βR is substituted by the LT-α2β1 complex that does not react with this receptor.

### Tumoricidal Activity of Peripheral Blood B Cells Is Deficient in HNSCC Patients

Previously we have shown that NK cells and DCs of tumor-bearing, treatment-naive HNSCC patients express decreased levels of TNFSF ligands and mediate lower tumoricidal activity relative to healthy blood donors (27). Since peripheral blood B cells of tumor bearing HNSCC patients also expressed decreased levels of the cytotoxic TNFSF ligands TNF, LT-β, FasL and TRAIL, we examined whether HNSCC patient B cells displayed diminished tumoricidal activity (**Figures 3C, D** and **Supplementary Figure 6**). We concurrently tested tumoricidal activity of sorted circulating B cells isolated from 4 healthy donors and 4 tumor-bearing, treatment-naive HNSCC patients against PCI-4a (**Figure 3C** **)** and PCI-13 (**Figure 3D** **)** HNSCC cell lines using the 24-h MTT assay. HNSCC patient B cells exhibited significantly lower cytotoxic activity than healthy donor B cells against both tumor cell targets.

### TNFSF Ligands Are Differentially Expressed in Blood and Tumor B Cell Subsets

**Figure 4:**
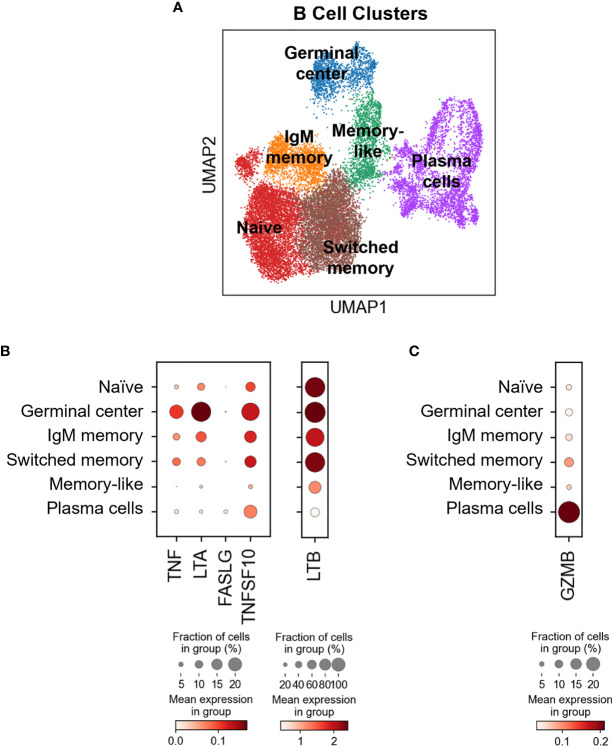
Differential TNFSF ligand and GZMB expression in different B cell subsets in blood and HNSCC tumors. **(A)** B cells identified in healthy donor and HNSCC patient blood and HNSCC tumors were extracted, subclustered and six different B cell differentiation states were identified using the curated list of genes listed in **Supplementary Figure 4B**. Dot-plots showing **(B)** TNFSF ligand and **(C)** GZMB expression by different B cell subsets. Color bars indicate normalized gene expression and dot sizes indicate the fraction of cells in a group expressing the particular gene.

Our flow cytometry data suggest that all the circulating B cells express transmembrane TNFSF ligands (**Figures 2A**, **3B**). To assess TNSF ligand transcript expression levels by various B cell subsets in blood and tumors, we reverted back to our scRNAseq dataset. Using a curated list of B cell-associated genes (*CD19*, *MS4A*/CD20, *SDC1*/CD138, *CR2*/CD21, *CD27*, *CD38*, *IGHD*/IgD, *SEMA4A*, *MKI67*/Ki67, *FCRL5*, *TNFRSF17* and *IRF4*) we identified at least six subtypes of B cells based on marker expression (**Figure 4A** and **Supplementary Figure 4B**): naive B cells (clusters 1, 4, 12,13, 14: *IGHD* ^+^ *CD38* ^-^), germinal center (clusters 7, 10; *CD38* ^+^ *SEMA4A* ^+^ *IGHD* ^low^), IgM memory (cluster 2: *CR2* ^+/-^ *CD27* ^low^ *IGHD* ^+^), switched memory (clusters 0, 9: *CD27* ^+^ *IGHD* ^-^), memory-like (cluster 3: *CD27* ^+^ *CD38* ^-^ *IGHD* ^-^) and plasma cells (clusters 5, 6, 8, 11: *CD19* ^+/-^ *MS4A1* ^-^ *SDC1* ^+^). The five TNFSF ligand transcripts were differentially expressed by all these subsets, with tumor-infiltrating germinal cells expressing the highest levels of *TNF*, *LTA*, *TNFSF10* and *LTB*, and tumor-infiltrating plasma cells expressing the highest levels of *FASLG* (**Figure 4B**). These observations indicate that tissue-resident B cell subsets, and particularly germinal center B cells, may have higher innate cytotoxic potential than those in circulation.

### Granzyme B Is Primarily Expressed by Tumor-Associated Plasma Cells

It has been reported that B cells can also express granzyme B (18). In this study, resting peripheral blood B cells were shown not to express granzyme B unless activated with IL-21 and/or by B cell receptor crosslinking (18). To exclude the possibility of granzyme B affecting the B cell-mediated cytotoxicity described in this manuscript, *GZMB* expression in different B cell subsets was evaluated by scRNAseq. It was revealed that plasma cells, which are primarily found in the tumor and not blood, express the highest levels of *GZMB*, sharply contrasting peripheral blood naïve and IgM memory B cells that express low levels of the transcript (**Figure 4C**). Switched memory B cells, that are found in both tumor and blood, displayed modest *GZMB* expression (**Figure 4C**). Cumulatively, these data suggest that granzyme B is predominantly expressed in the tumor and likely did not contribute to the killing mechanism described here. Of note, it was interesting to observe the divergence in the potential killing mechanisms by germinal center B cells (TNFSF ligands) and plasma cells (granzyme B), suggesting that these two cell types could mediate very specific cytotoxic functions within the tumor microenvironment.

## Discussion

A current notion is that immunosurveillance of newly generated cancer cells and their metastatic dissemination from a primary tumor is mediated by the innate immunity effectors, NK cells, while control of tumor growth is mediated by the adaptive immunity effectors, tumor-specific CD8^+^ cytotoxic T lymphocytes (CTLs) (28, 29). Both functions are thought to be mediated by engagement of specific receptors expressed on the effector cells that leads to consequent exocytosis of cytoplasmic granules and release of the cytotoxic molecules perforin and granzymes (30–32). However, the canonic secretory cytotoxic mechanism utilized by resting NK cells can only kill rare leukemia cell targets, and is largely ineffective against the majority of leukemia and virtually all solid tissue cancer cell types (1). Similarly, tumor-specific CTLs are rarely capable of inducing measurable growth inhibition or rejection of established tumors due to a variety of mechanisms (33, 34). On the other hand, the control of solid tumor growth in the absence of NK cells and CTLs has been described (35–39). Furthermore, in addition to NK cells and CTLs, CD4^+^ T cells, DCs and B cells have been found to variously colonize tumors and their increased frequency in tumor tissues correlates with favorable patient prognosis (40–43). Therefore, it is quite possible that other immune cell types and immune mechanisms might mediate ancillary immunosurveillance and antitumor activity.

In support of this idea, the present study newly defines that resting human peripheral blood B cells: 1) express multiple cytotoxic transmembrane TNFSF ligands such as TNF, FasL, LT-α1β2 and/or TRAIL; and 2) mediate a powerful innate apoptotic killing of virtually all types of leukemia and carcinoma cells and normal proliferating endothelial cells and fibroblasts through simultaneous engagement of these ligands. The last observation is of high importance as normal proliferating endothelial cells and fibroblasts are essential for tumor vasculogenesis and tumor stroma formation that are critical for tumor growth and metastasis (44, 45). Furthermore, as B cells and DCs are the major antigen presenting cells (10, 46), their apoptotic killing and fragmentation of cancer cells may be an initial step in tumor antigen uptake and processing that enables antigen presentation and leads to efficient induction of adaptive tumor specific immune responses. Therefore, this cytotoxic mechanism mediated by the major immune cells and their multiple TNFSF ligands could be central to both innate and adaptive immune control of tumor growth.

Our data indicate that resting B cells are particularly efficient killers of B and T leukemia cells and, therefore, may have a special role in immunosurveillance and elimination of aberrant and transformed B and T cells in lymphoid tissues. We also show that they kill cancer cells of primary tumors more efficiently than lymph node metastases, and may be critically involved in immunosurveillance of metastases, but could also participate in immunoediting of cancer cells. Finally, our scRNAseq data suggest that TNFSF ligands, which mediate this innate cytotoxic mechanism, are upregulated by tumor-infiltrating B cells, particularly by germinal center B cells. This is an important observation as transmembrane LT-α1β2, TNF and other TNFSF ligands also play a central role in the structural formation of secondary lymphoid organs and tertiary lymphoid structures (TLS) within tumors (47–49). By expressing and/or upregulating TNFSF ligands, B cells may also directly mediate the formation of primary and secondary follicles, germinal centers of secondary lymphoid organs and TLS in tumors. As these structures central to B cell-mediated immunity, through the activity of TNFSF ligands, B cells may bridge and optimize the innate and adaptive antitumor immune responses.

It is unclear how and why cancer cells escape this powerful immune mechanism and whether this function could be enhanced for cancer prevention and therapy. Cancer cells commonly upregulate sheddase activity of ADAM (a disintegrin and metalloproteinase) superfamily enzymes, such as ADAM17 and ADAM10 (50–53). These enzymes mediate cleavage of transmembrane TNFSF receptors, which leads to generation of high levels of soluble TNFSF receptors in tumor tissues and their consequent release into peripheral blood of cancer hosts (54, 55). As soluble TNFSF receptors specifically bind and potently neutralize TNFSF ligands, they can mediate negative regulation of TNFSF ligands. It is conceivable that this physiologic immunoregulatory mechanism is enhanced during both carcinogenesis and tumor growth. In support of this premise, it has been shown that both TNFSF ligand expression and apoptotic tumoricidal activity of NK cells and DCs were suppressed in tumor-bearing and restored in tumor-ablated patients with HNSCC (27). Additionally, soluble TNFSF receptors were increasingly bound to PBL of tumor-bearing HNSCC patients and their dissociation led to the restoration of TNFSF ligand expression and tumoricidal activity of NK cells and DCs (27). Our findings that the expression of cytotoxic TNFSF ligands and tumoricidal activity of B cells were suppressed in tumor-bearing treatment-naive HNSCC patients further support the tumor-dependent immunosuppressive mechanism. Therefore, targeted interventions that would simultaneously enhance the expression of transmembrane TNFSF ligands on the immune cells (i.e. their activation) and prevent cleavage of transmembrane TNFSF receptors on cancer cells (i.e. specific inhibition of ADAM superfamily members) could potentially enhance both the cancer preventive and therapeutic activities of the TNFSF ligand-mediated apoptotic tumoricidal activity of immune cells.

## Data Availability Statement

Unprocessed FASTQ files for our published scRNAseq datasets (20, 21) are available on NCBI Sequence Read Archive (BioProject ID PRJNA579178 and PRJNA691564). Processed gene barcode matrices are available on the Gene Expression Omnibus database (Accession ID GSE139324 and GSE164690).

## Ethics Statement

The studies involving human participants were reviewed and approved by University of Pittsburgh Institutional Review Board. The patients/participants provided their written informed consent to participate in this study.

## Author Contributions

LV and NV were accountable for the conception and design of research and writing the manuscript. LV, BJ and NV performed all *in vitro* experiments and data analyses. AK performed scRNAseq analyses, and RLF provided HNSCC patient PBMC and contributed to writing the manuscript. All authors contributed to the article and approved the submitted version.

## Funding

This work was supported by research funding from National Institutes of Health grants I-PO DE13059 and RO1 DE14775 (NV.). This study was also supported in part by the UPMC Hillman Cancer Center Head & Neck Cancer SPORE (NIH P50 CA097190; PI Ferris/Grandis) Developmental Research Program award (LV) and the UPMC Hillman Cancer Center Skin SPORE (NIH P50 CA121973; P.I. Kirkwood) Developmental Research Program award to LV.

## Conflict of Interest

The authors declare that the research was conducted in the absence of any commercial or financial relationships that could be construed as a potential conflict of interest.

## Publisher’s Note

All claims expressed in this article are solely those of the authors and do not necessarily represent those of their affiliated organizations, or those of the publisher, the editors and the reviewers. Any product that may be evaluated in this article, or claim that may be made by its manufacturer, is not guaranteed or endorsed by the publisher.
