# Supplementary material for: Human B Cells Mediate Innate Anti-Cancer Cytotoxicity Through Concurrent Engagement of Multiple TNF Superfamily Ligands
Source: Front Immunol. 2022 Mar 22;13:837842. doi: 10.3389/fimmu.2022.837842 (PMC8983021; doi:10.3389/fimmu.2022.837842)
Supplement: Supplementary file 1 [file DataSheet_1.pdf]

Supplementary Table 1. Demographics of 29 HNSCC patients evaluated by scRNAseq.

		Number (%)
Sex	Male	23 (79.3%)
	Female	6 (20.7%)
Mean age (years)		59.5
Race	Caucasian	29 (100%)
Primary Site	Larynx	2 (6.9%)
	Oral cavity	17 (58.6%)
	Oropharynx	10 (34.5%)
p16 status	Negative	18 (62.1%)
	Positive	11 (37.9%)
Stage Group*	I	6 (20.7%)
	II	6 (20.7%)
	III	5 (17.2%)
	IVA	10 (34.5%)

Supplementary Table 2. HNSCC metastatic cell lines are less susceptible to DC or B cell-mediated killing than those from matching primary tumors

	$LU_{20}/10^7$ Effector cells	
Target Cells	DCs	B cells
PCI-4A	23977	21449
PCI-4B	14906	2252
PCI-6A	6224	6244
PCI-6B	932	2303
PCI-15A	5743	12447
PCI-15B	2661	3965
PCI-22A	6301	7695
PCI-22B	598	6755
PCI-13	4952	1822
PCI-30	1009	518
PCI-37A	3735	6060
HUVEC	1074	650
Keratinocytes	0	0

A, primary tumor; B, metastasis; Normal cells are in Italic.

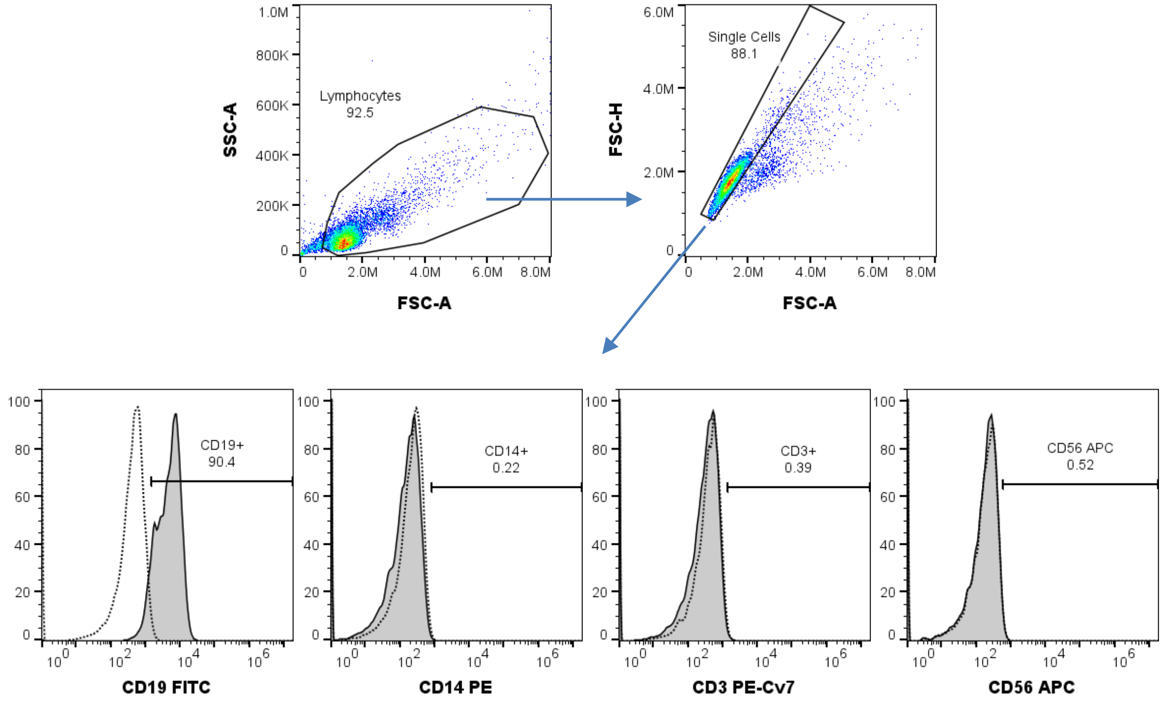

Supplementary Figure 1. MACS-sorted circulating B cells have ≥ 90% purity based on flow cytometric phenotyping. B cells were sorted from donor PBL by MACS per ***Materials and Methods***. Lymphocytes were gated based on size (FSC), granularity (SSC) and single-cell status (FSC-A vs FSC-H). Subsequently, cells were evaluated for CD3, CD14, CD19 and CD56 expression. Data are representative of three staining experiments. Dotted lines – IgG controls; filled histograms – specific markers.

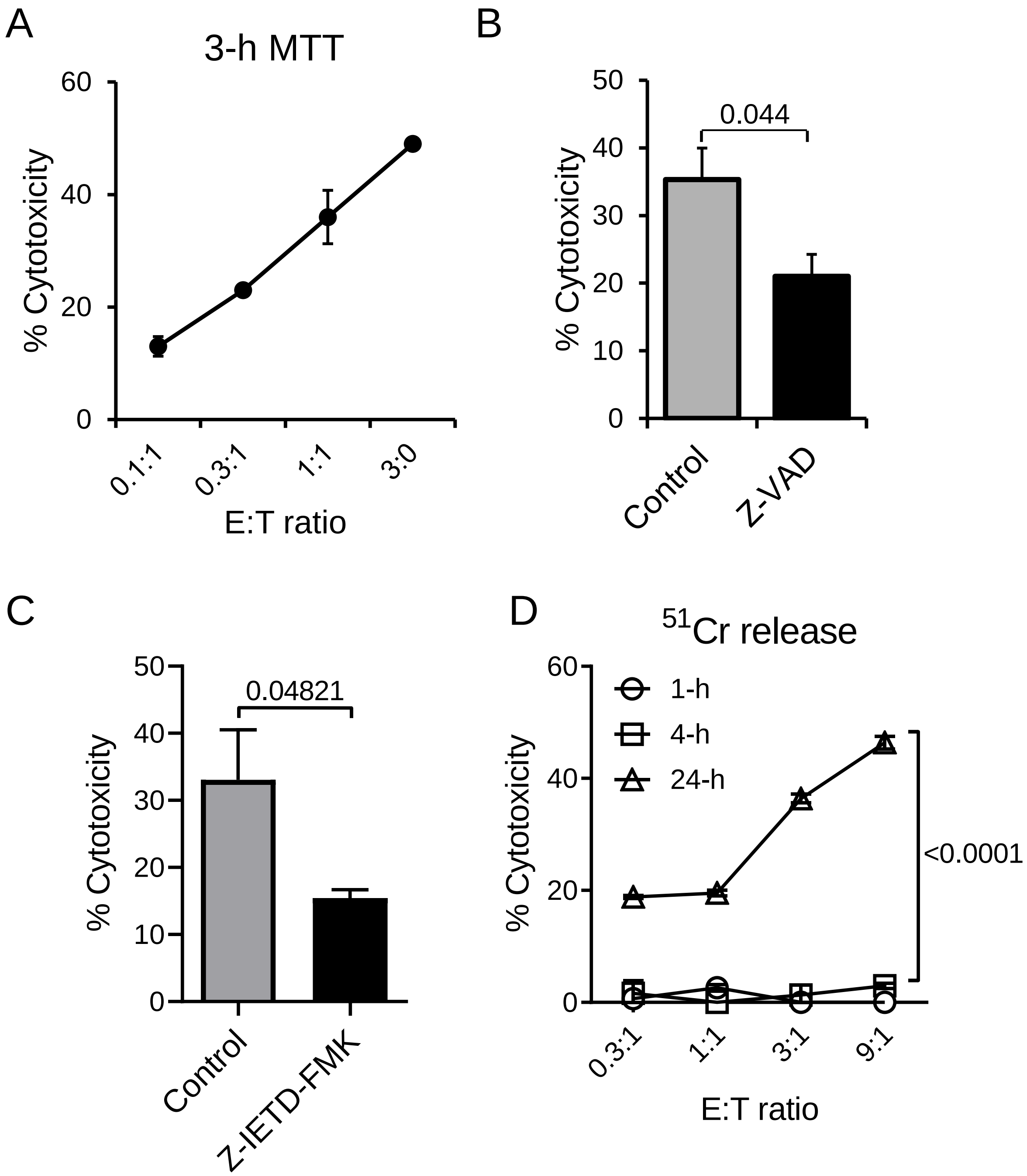

Supplementary Figure 2. Resting B cells induce apoptosis in tumor targets. MACS-purified healthy donor B cells were used as effectors and PCI-13 HNSCC cells as targets at the indicated effector-to-target (E:T) ratios. **(A)** We validated the 3-h MTT assay as means to evaluate B cell-mediated killing of tumor targets. Data represent means of percentages of cytotoxicity ± standard deviation (STDEV) obtained from 4 replicates of a representative experiment of 3 performed. **(B-C)** To evaluate whether caspases are involved in B cell-mediated killing of cancer cells, PCI-13 cancer cells were preincubated with **(B)** the pan-caspase inhibitor, Z-VAD-FMK, (50 mM) or **(C)** the caspase 8-specific inhibitor, Z-IETD-FMK (100 mM), for 1 h, co-incubated with B cells at 1:1 E:T ratio in 4-6 replicates. The cytotoxic activity was assessed using **(B)** 3-h and **(C)** 24-h MTT assays. Data are means ± STDEV. **(D)** 1-h, 4-h and 24-h 51Cr release assays were used to measure B cell-induced necrosis of tumor targets. Data are summarizing two independent experiments. Each test was performed in 4 replicates. Mean percent cytotoxicity values ± STDEV of the two experiments are shown.

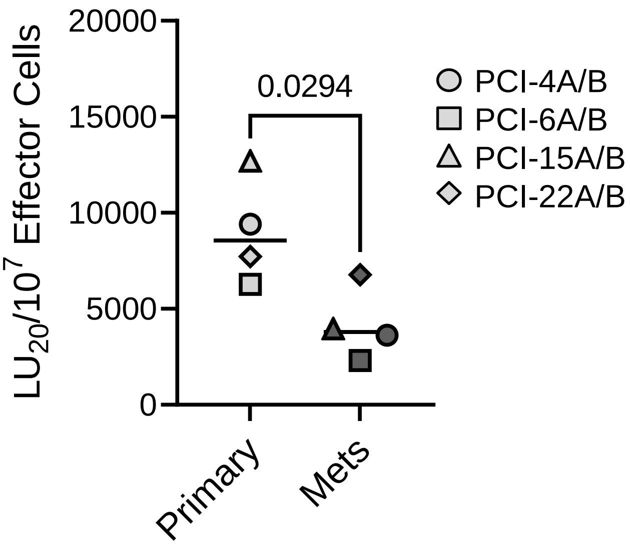

Supplementary Figure 3. Cancer cells of lymph node metastases are less susceptible to killing by resting B cells than cancer cells of primary tumors. Four pairs of primary tumor and lymph node metastasis cancer cells (labeled as “A” and “B,” respectively) obtained from 4 different HNC patients were tested in parallel for their susceptibility to killing by resting circulating B cells using the 24-h MTT assay. Data represent individual donor $LU_{20}/10^7$ effector cells and their means (horizontal lines) based on the percentages of cytotoxicity obtained at 4 E:T ratios. Tests were performed in 4 replicates. *P*-value shown is calculated using the one-tailed student *t*-test.

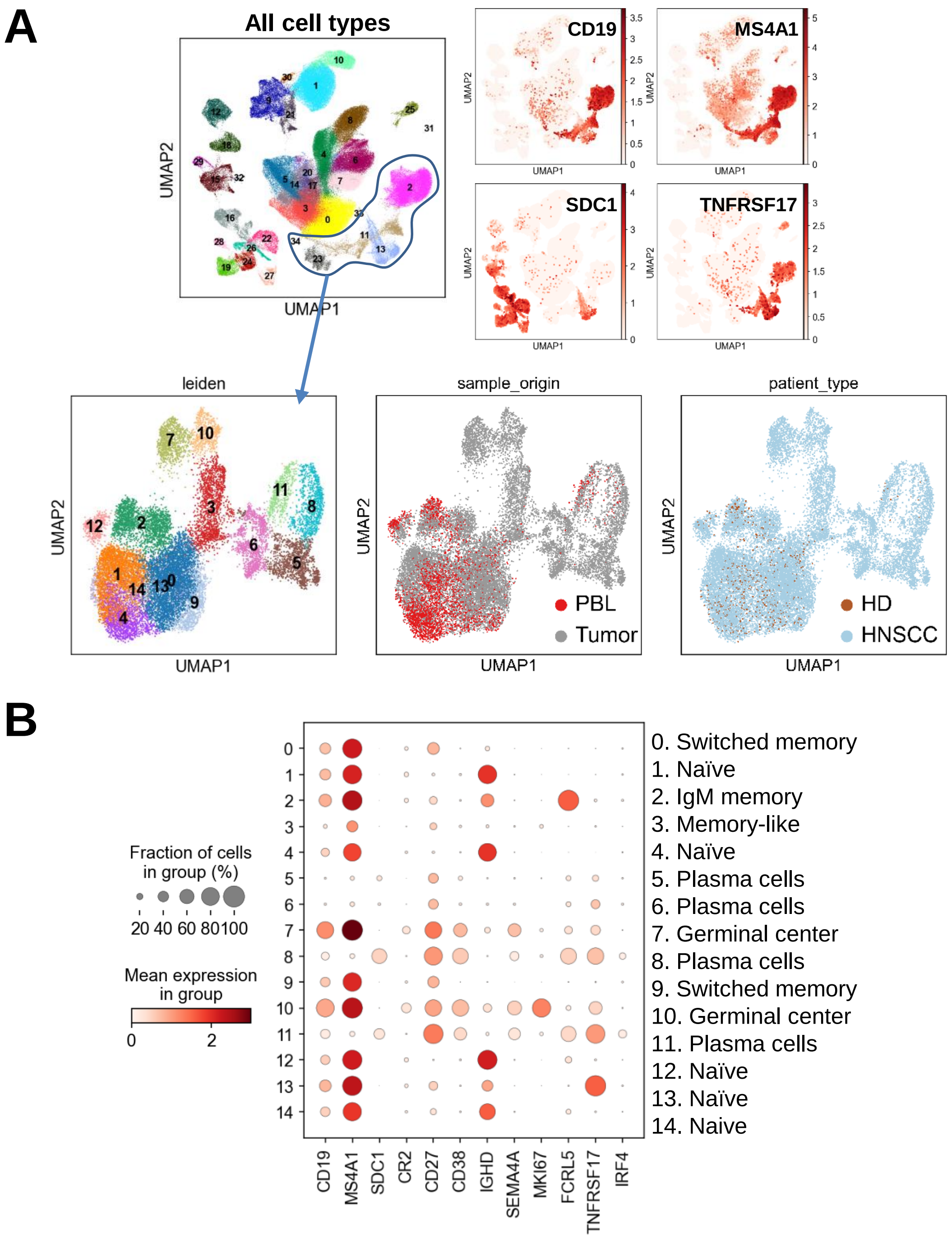

Supplementary Figure 4. Identification and visualization of circulating B cells isolated from our previously published scRNAseq datasets. Our published scRNAseq datasets from a cohort of 6 healthy donors and 29 treatment-naïve HNSCC patients were used to evaluate circulating B cell profiles. **(A)** B cell populations were identified using canonical B cell lineage markers, CD19 and MS4A1 (CD20), and by canonical plasma cell markers, SDC1 (CD138) and TNFRSF17 (B cell maturation antigen). Once identified, B cell populations were extracted and further subclustered. UMAP dimensionality reduction of B cells was performed based on cell clusters (0-14), tissue of origin [PBL vs tumor] and donor health status [healthy donors (HD) vs. HNSCC patients (HNSCC)]. **(B)** Dot plot showing expression of marker genes used for cell type assignments depicted in **Fig. 4**.

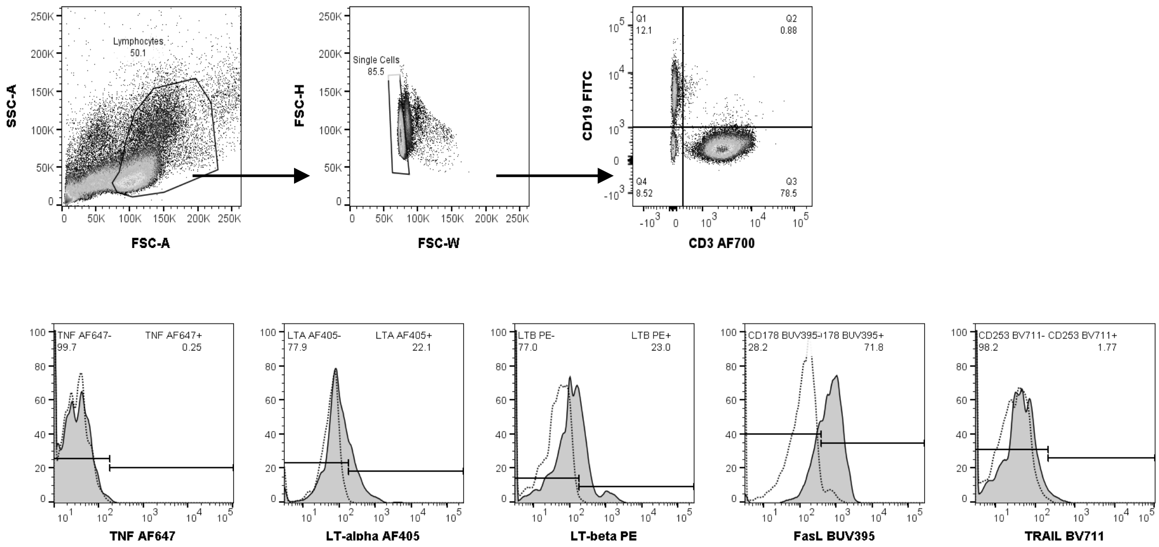

Supplementary Figure 5. Flow cytometric gating strategy used to evaluate circulating B cells for TNFSF ligands. PBL were stained for flow cytometry per ***Materials and Methods***. Lymphocytes were gated based on their FSC vs SSC profile and single-cell status. Fluorescence minus five (FM5) controls labeled with CD3 AF700 and CD19 FITC, as well as control IgG antibodies for AF647, AF405, PE, BUV395 and BV711 antibodies, were used to set upper limits for background signals on the missing labels. $CD19^{+}$ $CD3^{-}$ B cells were analyzed for TNF AF647, LT-α AF405, LT-β PE, FasL BUV395 and TRAIL BV711 surface staining. Representative data from one of the five HNSCC patients evaluated are shown. Dotted lines on histograms represent FM5 IgG controls, and filled histograms represent the actual staining. Data presented in **Fig. 3** are standardized against their corresponding FM5 IgG controls.

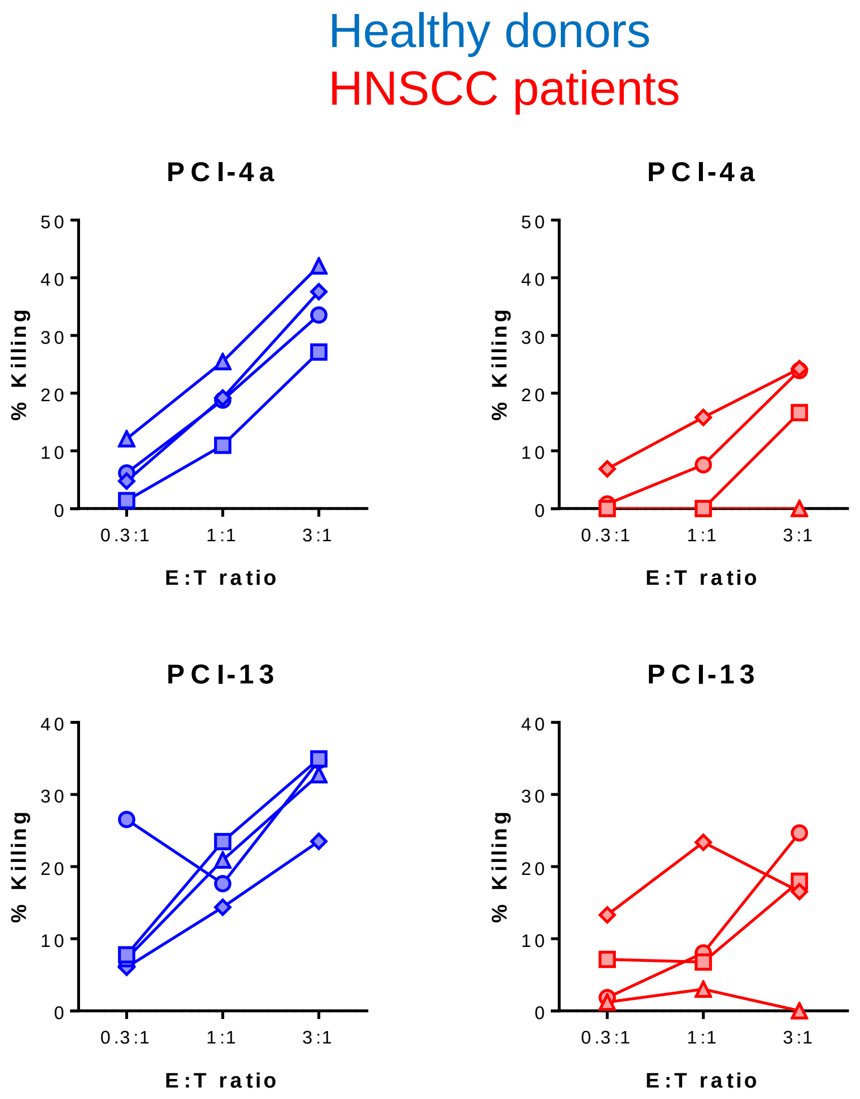

Supplementary Figure 6. B cell tumoricidal activity of HNSCC patients is deficient. Cytotoxic activities of B cells of individual healthy donors and HNSCC patients (n = 4) are shown. Experiments were performed as described in the legend for **Fig. 3**. Data are mean percentages of cytotoxicity of 4 replicates.
